# Tracking the Molecular Fingerprint of Head and Neck Cancer for Recurrence Detection in Liquid Biopsies

**DOI:** 10.3390/ijms23052403

**Published:** 2022-02-22

**Authors:** Araceli Diez-Fraile, Joke De Ceulaer, Charlotte Derpoorter, Christophe Spaas, Tom De Backer, Philippe Lamoral, Johan Abeloos, Tim Lammens

**Affiliations:** 1 Division of Oral and Maxillofacial Surgery, Department of Surgery, General Hospital Sint-Jan Brugge-Oostende A.V., 8000 Bruges, Belgium; araceli.diez-fraile@azsintjan.be (A.D.-F.); joke.deceulaer@azsintjan.be (J.D.C.); christophe.spaas@azsintjan.be (C.S.); tom.debacker@azsintjan.be (T.D.B.); philippe.lamoral@azsintjan.be (P.L.); johan.abeloos@azsintjan.be (J.A.); 2 Department of Pediatric Hematology-Oncology and Stem Cell Transplantation, Ghent University Hospital, 9000 Ghent, Belgium; charlotte.derpoorter@ugent.be; 3 Department of Internal Medicine and Pediatrics, Ghent University, 9000 Ghent, Belgium; 4 Cancer Research Institute Ghent (C.R.I.G.), 9000 Ghent, Belgium

**Keywords:** head and neck squamous cell carcinoma, surveillance, liquid biopsy, minimal residual disease, relapse

## Abstract

The 5-year relative survival for patients with head and neck cancer, the seventh most common form of cancer worldwide, was reported as 67% in developed countries in the second decade of the new millennium. Although surgery, radiotherapy, chemotherapy, or combined treatment often elicits an initial satisfactory response, relapses are frequently observed within two years. Current surveillance methods, including clinical exams and imaging evaluations, have not unambiguously demonstrated a survival benefit, most probably due to a lack of sensitivity in detecting very early recurrence. Recently, liquid biopsy monitoring of the molecular fingerprint of head and neck squamous cell carcinoma has been proposed and investigated as a strategy for longitudinal patient care. These innovative methods offer rapid, safe, and highly informative genetic analysis that can identify small tumors not yet visible by advanced imaging techniques, thus potentially shortening the time to treatment and improving survival outcomes. In this review, we provide insights into the available evidence that the molecular tumor fingerprint can be used in the surveillance of head and neck squamous cell carcinoma. Challenges to overcome, prior to clinical implementation, are also discussed.

## 1. Introduction

Head and neck malignancies encompass a diverse group of aggressive tumors arising at the epithelium of the upper aerodigestive tract mucosal linings, squamous cell carcinoma being the most frequent histopathological type (90%). Head and neck squamous cell carcinoma (HNSCC) has been associated with external risk factors such as smoking habits, alcohol consumption, and infection with herpes papillomavirus (HPV) or Epstein-Barr virus (EBV), all of which are reflected in the genomic and transcriptomic profiles of their respective factor-based subtypes [1,2,3,4,5]. Furthermore, genetic predisposition has been identified as a risk factor for developing head and neck cancers in a growing number of patients [6,7].

The 5-year relative survival rate for head and neck tumors improved from 53% in the 1970s to 67% in the 2010s in the USA [8]. Despite aggressive treatment with surgery, radiotherapy, chemotherapy, photodynamic therapy, immunotherapy, or any combination thereof, approximately 30% of patients will relapse within two years of treatment; relapse is usually associated with poor outcomes [9].

Monitoring minimal residual disease and disease dynamics is of paramount importance to the timely detection of recurrence, as awareness when the tumor burden is minimal can maximize the efficacy of salvage treatment and improve survival [10,11]. Currently, there is a lack of consensus on the optimal surveillance method, and practices vary widely across clinicians and institutions. Conventional tools include clinical examination supported by endoscopy and/or imaging modalities such as positron emission tomography-computed tomography (PET/CT) and magnetic resonance imaging (MRI), followed by biopsy when a suspect mass is observed. However, clinical examination cannot detect lymph node micrometastases, and imaging techniques are costly and challenging, particularly in the elderly population due to the presence of prosthetic and implant metallic streaking artifacts, but also owing to post-operative fibrosis and treatment-induced inflammation [12,13]. Additionally, biopsies are invasive, time-consuming procedures that are difficult to repeat, and do not reflect the spatiotemporal heterogeneity of a solid tumor because they target only a single tumor site at a specific time point. Hence, the identification of an optimal follow-up approach for the detection of recurrent pathology in a timely and cost-effective manner is imperative.

**Figure 1:**
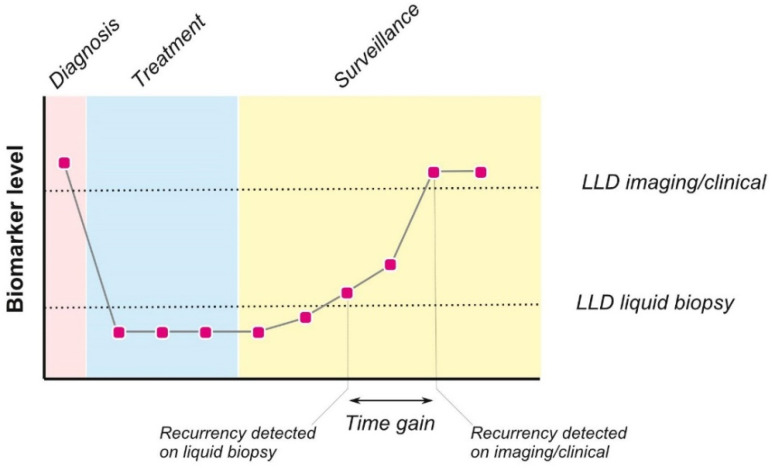
Liquid biopsy profile versus imaging/clinical surveillance. Hypothetical patient surveillance curves using one or several liquid biopsy markers as discussed (black line). Dotted lines indicate the threshold levels which would allow imaging/clinical or liquid biopsy-based surveillance to trigger the suspicion of recurrence. Indicated is the hypothetical time gained using liquid biopsy-based surveillance, allowing faster clinical interventions.

**Figure 2:**
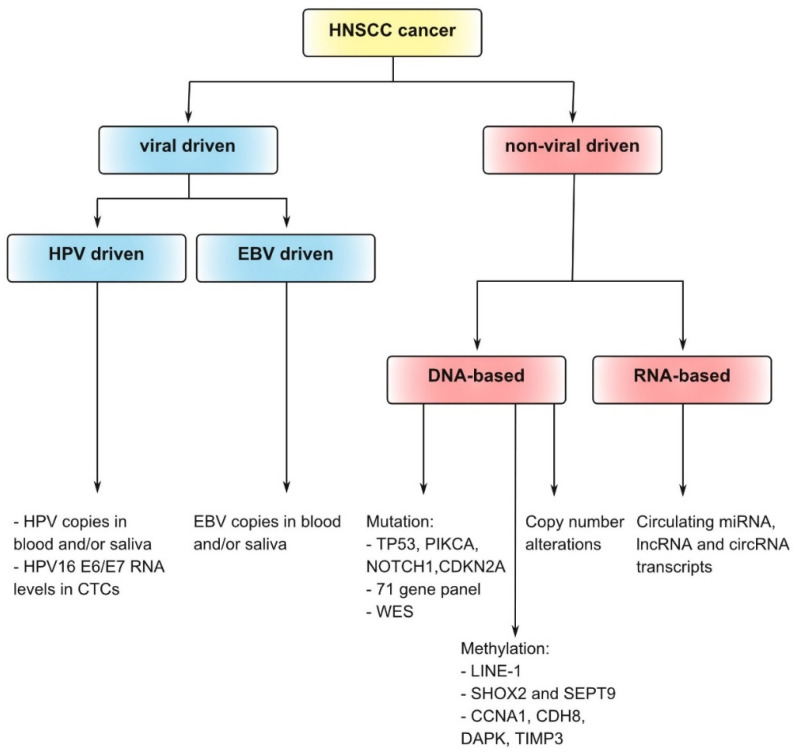
Liquid biopsy surveillance strategies for head and neck squamous cell carcinoma (HNSCC). Summary of liquid biopsy-based surveillance strategies as discussed throughout this review. Although several choices are indicated (i.e., viral- or non-viral-driven tumors), it should be noted that a combination of strategies/surveillance options would probably offer the optimal path forward. RNA-based options are scarcely represented in the literature but are indicated as an option in this diagram.

Recent studies have demonstrated that body fluid-based diagnostic methods offer real-time monitoring during follow-up (Figure 1), aiding in early detection of tumor recurrence, treatment resistance, and therapeutic decision-making [14,15]. These methods offer substantial advantages over conventional surveillance-based methods, including cost efficiency and minimal invasiveness, which improve the potential for repeated analyses and higher patient compliance. Liquid biopsy analytes that have generated considerable interest include cell-free circulating tumor (ct) DNA, ctRNA, circulating proteins, metabolites, platelets, circulating tumor cells, and tumor-derived extracellular vesicles [14,15]. In addition to different analyte types, the number of methods to study and evaluate these analytes has rocketed. Next to standard techniques available in most diagnostics laboratories, innovative high-end exploratory methods have been used, including microarray analysis and (single-cell) sequencing and proteomic approaches [16,17,18,19,20,21]. The power and utility of liquid biopsies for HNSCC have recently been shown, capturing the complex and dynamic features of the disease. Besides blood, upper aerodigestive secretions such as saliva have also been used to reveal information about HNSCC [22,23,24]. Here, we summarize the current evidence on the use of tumor-biology-driven biomarkers using ctDNA and ctRNA for HNSCC surveillance (Figure 2). We further suggest future directions for the integration of liquid biopsy-based approaches for HNSCC cancer surveillance into clinical practice.

## 2. ctDNA as Liquid Biopsy Markers Suitable for Longitudinal Surveillance in HNSCC

ctDNA consists of DNA fragments that are released from tumor cells into biological fluids including blood, saliva, cerebrospinal fluid, and urine [25]. While ctDNA released from the basal side of HNSCC epithelial cells has been detected in plasma samples, DNA released on the apical side of cancerous cells is detectable in the saliva [23,26,27]. Recent reports in other tumor types clearly illustrate that ctDNA offers the possibility to capture tumor-derived genetic and epigenetic alterations in liquid biopsies during post-treatment surveillance of malignant tumors [28,29]. Below we will discuss the current state-of-the-art of viral and carcinogen-driven HNSCC detection using ctDNA.

### 2.1. Detection of HPV ctDNA in Biologic Fluids

Determining HPV status is of critical importance in the diagnosis and management of HNSCC, as HPV-positive tumors have unique pathological and clinical characteristics with implications for prognosis and treatment decisions. Generally, HPV-positive tumors are susceptible to radiation and anticancer drugs, and thus have a better prognosis (5-year overall survival: 75–80%) as compared to HPV-negative tumors [30]. This improved survival rate is also driven by a simpler mutational load, since the tumor suppressor genes TP53 and RB1 are silenced by viral oncoproteins E6 and E7 [31]. Nevertheless, activating mutations of genes involved in the PI3K pathway are frequently observed [31]. Most high-risk HPV-induced HNSCC affects the oropharyngeal region and presents at a high stage, often metastasized to nearby lymph nodes. The incidence of HPV-related head and neck cancer is currently growing among a relatively young population [1,4]. HPV presence is generally evaluated in a tissue sample at diagnosis, using quantitative PCR of HPV16 E6 and E7 oncoproteins or by immunohistochemistry. Several studies have pointed to the usefulness of HPV16 DNA analysis in saliva and plasma as a post-treatment surveillance tool [22,23,24]. In a small cohort of HPV-positive patients (n = 20), Chuang and colleagues [22] showed that recurrence could be identified in follow-up saliva rinses done every three months until two years after treatment. This study illustrated that detection of HPV16 copy numbers in follow-up saliva rinses was positive in half of the patients presenting with a recurrence (2/4; sensitivity of 50%) and in none of the patients without recurrence (specificity of 100%). Importantly, the presence of HPV ctDNA in saliva was already noted at three months after treatment completion (for patients eventually relapsing; n = 2), whereas disease-free survival was for each patient 16.5 and 12 months from completion of therapy as per standard clinical evaluation [22]. In addition to evaluating saliva samples, Ahn and colleagues [23] showed in a retrospective study of 81 HPV-positive HNSCC patients that the combined determination of HPV16-DNA status in blood and saliva samples post-treatment was highly specific (90.7%) and sensitive (69.5%) for predicting recurrence within 3 years. Although the combined study of blood and saliva provides, in general, the best specificity and sensitivity, for HPV-associated tumors located mainly at the oropharynx, plasma is more informative than saliva (sensitivity of 86% vs. 40%, respectively) [24]. In addition to the type of fluid, the technique used has also been shown to impact the results. Droplet digital PCR (ddPCR) represents the most sensitive and accurate mode of HPV detection in oropharyngeal squamous cell carcinoma (OPSCC) without a tissue biopsy. ddPCR of oropharyngeal swabs was performed with a sensitivity of 92% and specificity of 98% calculated against p16 IHC [32].

Recently, Chera and colleagues performed a prospective biomarker trial of patients with non-metastatic HPV-associated (p16-positive) OPSCC [33]. Participants underwent extensive post-treatment imaging and clinical surveillance in addition to plasma-based HPV DNA analysis every 6–9 months. This controlled trial clearly illustrated that the detection of circulating HPV-DNA in two consecutive plasma samples during post-treatment surveillance had both a high positive predictive value (>95%) as well as a high negative predictive value (100%) for identifying disease recurrence in patients with HPV-associated OPSCC. These results facilitated earlier initiation of salvage therapy in patients with recurrence and formed the basis of the NavDx^®^ DNA blood test currently available in the United States (https://naveris.com/what-is-navdx/, accessed on 23 December 2021). The company is currently working on a saliva-based version of the test, for which results matching those of the blood-based assay were presented at the American Society of Clinical Oncology 2021 annual meeting [34].

### 2.2. Analysis of EBV ctDNA during HNSCC Surveillance

Several studies have detected the presence of EBV in HNSCC in specific regions of the world (Southern China and Southeast Asia), implying its possible role in the development of malignancies throughout the upper aerodigestive tract, with most cases presenting at the nasopharynx [35,36]. The EBV-encoded oncoproteins EBNA1, LMP1, and LMP2 control the carcinogenic properties of the virus, from oncogenesis to progression and invasion of human cancer cells via the induction of epithelial-to-mesenchymal transition [37,38].

Similar to HPV, EBV ctDNA has also been recognized as a promising biomarker with the potential for accurate early detection of nasopharyngeal carcinoma (NPC) recurrence [39,40,41,42,43,44,45]. Specifically, detectable EBV ctDNA in plasma during follow-up indicates tumor recurrence, while undetectable EBV ctDNA indicates continuous remission [39,41,43,44]. A recent study based on a large cohort of NPC patients (n = 1984) indicated that sequential analyses of post-treatment EBV ctDNA in blood, using qPCR for the BamHI-W region of the EBV genome, predicted relapse with a sensitivity and specificity of 82.3 (79.0–85.3%) and 80.0% (77.8–82.1%), respectively [44]. Importantly, these results varied according to the site and type of recurrence, with the best performance noted for non-pulmonary sites and distal metastasis [44]. Among the patients with detectable EBV ctDNA who experienced disease recurrence, the positive EBV ctDNA results preceded radiological and/or clinical evidence of disease recurrence by a median of 2.3 months (interquartile range 0.1–9.5 months). Finally, Wang and colleagues have suggested that post-surveillance screening based on plasma EBV ctDNA be used to recommend PET screening for high-risk patients. This could result in cost savings of approximately 80% by performing 2–4 blood tests per year instead of an annual PET [41]. The latter was recently confirmed by a model-based analysis of surveillance strategies in NPC, showing that EBV ctDNA-guided imaging strategies for stage II–IV patients are considerably more economical than routine imaging strategies [46].

In line with these reports and given that up to 66% of recurrences are being missed when following the previous National Comprehensive Cancer Network and European Society for Medical Oncology (ESMO) guidelines, the most current ESMO guidelines published in 2020 recommend evaluating EBV ctDNA at least every year [45,47].

### 2.3. ctDNA in Carcinogen-Driven HNSCC

Gene- and pathway-focused sequencing initiatives have shed light on our understanding of the molecular pathogenesis of non-viral-driven HNSCC. These studies have shown that non-viral HNSCC is broadly driven by non-mutually exclusive mutations in genes involved in four pathways: cell survival and proliferation (TP53, EGFR, PIK3CA, and HRAS), cell-cycle control (CDKN2A and CCND1), cellular differentiation (NOTCH1), and cellular adhesion and invasion signaling (FAT1) [31,48,49]. Mutations in TP53, CDKN2A, FAT1, PIK3CA, NOTCH1, KMT2D (MLL2), NSD1, CASP8, AJUBA, and NFE2L2 genes have been frequently reported in HNSCC [31].

The groundwork for surveillance clinical tests for the early detection of HNSCC recurrence based on tumor-shed DNA mutations was laid by Wang and colleagues [24]. These researchers evaluated the presence of ctDNA in blood and/or saliva samples of 93 patients before surgery, as well as for nine patients at various time points after surgery. First, tumors were assessed for HPV positivity. HPV-negative tumors were then screened for TP53, PIKCA, NOTCH1, and CDKN2a mutations; if none of those regions of interest were mutated, then a low-pass sequencing approach was used to identify any somatic tumor mutations or rearrangements [24]. These findings were subsequently used to screen for HPV ctDNA or somatic tumor mutations in saliva and/or blood. They concluded that the use of both compartments (blood and saliva) allowed, in a large heterogeneous cohort of tumors, for the detection of ctDNA and alterations in 96% of the patients pre-surgery. Moreover, the authors observed that sensitivity for the detection of ctDNA in saliva was dependent on the location of the primary tumor (the test was more effective in tumors of the oral cavity) and on the disease stage (saliva was more sensitive in early stages, and plasma in late). The authors identified ctDNA in the saliva of patients whose tumors were not clinically measurable until 9–19 months later, suggesting that these tests could provide a clinically meaningful lead time for referral to close follow-up and/or initiation of salvage treatment. Several other reports have confirmed the clinical utility of liquid biopsies for mutation-based detection of HNSCC recurrence, albeit with varying levels of sensitivity [26,50,51,52,53].

As whole-genome sequencing becomes more accessible, large sequencing efforts such as The Cancer Genome Atlas initiative have been key for detecting additional alterations specific to the pathogenesis of HNSCC tumors. Some genes that have been identified this way include members of the TRK family (EGFR, ERBB3, AXL, CSF1R, and RET), MAPK pathway (MAPK1 and NF1); genes implicated in chromatin regulation (KMT2C, KMT2B, NSD1, CREBBP, SMARCA2, and SMARCA4); genes of the NOTCH, Hedgehog, and Wnt pathways; apoptosis genes (CASP8); and genes involved in DNA repair (ARID1A, BRCA1, MSH2, and MLH1) [31,54]. The TCGA-HSNCC data, accessible at the Genomic Data Commons Data Portal [55], illustrates the top 20 mutated genes (TP53, FAT1, CDKN2A, PIK3CA, NOTHC1, KMT2D, NSD1, CASP8, FAT4, KMT2C, HRAS, CREBBP, FBXW7, AKAP9, NFE2L2, RNF213, SPN, SOS1, and RUNX1T1) in 528 HNSCC cases, which might serve as a highly relevant panel for targeted screening. This has not only enabled the detection of actionable (druggable) alterations and captured the heterogeneity of HNSCC tumors, but also broadened the mutational panel for surveillance. The increased understanding of the pathways and genes involved in HNSCC tumorigenesis could lead to a higher clinical sensitivity of future panels and allow for more confidence in distinguishing relapse from second primary tumors.

Recently, Cui and colleagues [56] improved on a panel of genes implicated in HNSCC. Tumor-specific mutations were identified in a study on oral squamous cell carcinoma (OSCC) using whole-exome sequencing, and frequent somatic mutations were searched for in TCGA and COSMIC to yield a final panel of 71 frequently mutated genes involving oral cancer, optimized for HNSCC. Their study involved 11 patients from whom plasma and saliva were collected for six months after surgery; clinical recurrence follow-up was done for 18 months after surgery. Targeted deep-sequencing was employed to detect mutations in the different liquid biopsy samples. Interestingly, five out of six relapses were detected in ctDNA saliva analyses before any clinical sign of recurrence, and additional plasma sampling confirmed the remaining case. Moreover, recurrence was detected on average 4.4 months earlier using ctDNA analysis as compared to imaging (CT- or MRI-based) or clinical examination (naked-eye visual inspection or palpation). For OSCC, it appears that saliva-based lab monitoring is able to capture most recurrences at a very early phase. In addition, this study illustrates the strength of generating patient-specific panels.

In an effort to encompass as many tumor-related alterations as possible, Mes and colleagues [57] analyzed copy number alterations (CNAs) along with tumor mutations. Indeed, HNSCC is frequently characterized by losses of 3p, 9p, and 17p, and gains of 3q, 7p, and 8q [58,59,60]. Importantly, the combined analysis of CNAs and somatic mutations in patients with known tumor DNA aberrations revealed tumor-associated aberrations in plasma DNA in 21 out of 27 patients (78%), while either analysis alone performed less well (67% for can analysis, 52% for mutation profiling) [57]. This illustrates a clear benefit of combined analysis.

As compared to the detection of HPV or EBV ctDNA sequences after cancer treatment, specific genetic lesions are not as specific for predicting tumor recurrence. For example, pathogenic mutations for TP53 have been reported in oral rinses from both cancer patients and non-cancer controls [50], thereby challenging the significance of ctDNA in surveillance tests for HNSCC. Since early driver TP53 mutations show high concordance between primary and recurrent and/or metastatic tumors, they may hold promise as reliable targets for early detection of HNSCC recurrence [52,61]. However, the cancer’s somatic origin should be initially verified, by demonstrating its absence in the germline, before the design of any subsequent ctDNA assays.

In addition to mutations and CNAs, methylation of circulating DNA has also been employed as a liquid biomarker. DNA methylation is an epigenetic mechanism of gene expression control, and its dysregulation has been reported in various cancers, including HNSCC. Methylation signatures of circulating DNA have proven useful in the detection and classification of different cancer types at diagnosis [29,62], and methylation signatures of diagnostic tissue samples provide prognostic value for HPV-positive head and neck cancers [63,64]. For example, the transposable element LINE-1 has been shown to be hypermethylated in HPV-positive HNSCC tumors [64], depending on its location in the genome (with intergenic regions being hypermethylated). Because it would be present in multiple cell-free copies in blood, LINE-1 methylation would likely outperform single-copy genes in ctDNA liquid biopsy [64]. However, direct evidence that LINE-1 activation can be used as a cancer biomarker is still limited. Finally, DNA methylation signatures are capable of discriminating HPV-positive and HPV-negative HNSCC [65,66].

Aberrantly methylated copies of the SHOX2 and SEPT9 genes are among the liquid biopsy methylation biomarkers with the highest level of validation regarding their clinical performance [67]. After establishing the diagnostic potential of these methylated copies, de Vos and colleagues [68] evaluated their performance in monitoring disease recurrence in comparison to clinical examination or CT scan monitoring (n = 8). Four of these cases showed progressive disease. Importantly, in only one case did methylation scoring in ctDNA increase earlier than the detection of progressive disease. Similarly, Machado de Jesus and colleagues [69] used ddPCR to quantify CCNA1, CDH8, DAPK, and TIMP3 promoter methylation in pretreatment formalin-fixed paraffin-embedded (FFPE) biopsies (n = 52) and paired plasma (n = 20) samples. Concordant results were found between both sample types in 16 out of 20 patients, with 11 out of 15 FFPE methylation-positive patients also positive in pretreatment plasma and full concordance in negative cases (n = 5). In three samples with detectable CCNA1 methylation in the pretreatment plasma samples, post-treatment plasma was also available. Interestingly, one patient had no decrease in CCNA1 methylation that could be observed at day 414 after treatment completion and was subsequently diagnosed with a cervical lymph node metastasis. In a second patient, increased methylated CCNA1 was observed at day 195 after treatment completion and they were subsequently diagnosed with a local recurrence at day 518 post-treatment.

## 3. The Value of ctRNA in Cancer Monitoring

Multiple studies have explored the coding and non-coding transcriptome of HNSCC cancers in a systematic manner [70,71,72]. These studies have illustrated the importance of ZEB2-driven epithelial-to-mesenchymal transition, cell cycle genes (E2F, CDK4/6), and immune-related expression of PD-L1 and CTLA-4 as key drivers of HNSCC pathogenesis [31,73,74,75]. In addition, deregulated expression of microRNAs and long non-coding RNAs has recently been documented in HNSCC tumor material, impacting the expression of genes involved in invasion and metastasis, as well as that of MDM2 involved in the TP53 program [76,77,78].

Tumor-derived RNA has been identified in different body fluids (e.g., blood, saliva), and could serve as a biomarker for disease diagnosis and stratification, detection of minimal residual disease, and determination of prognosis. The past decade has produced myriad reports describing blood- and saliva-based RNA expression in HNSCC. For HPV-positive tumors, HPV16 E6/E7 ctRNA expression in circulating tumor cells has proven useful as a biomarker for clinically relevant HPV infection in HPV-positive OPSCC [79]. In addition, circulating microRNA and long non-coding RNA expression has been recently reviewed [80]. Finally, circular RNAs have also gained attention. Shuai and colleagues [81] illustrated how circ_0000285 expression in the serum of NPC patients was correlated with tumor size, degree of differentiation, lymph node metastasis, distal metastasis, and TNM stage, and the level of circ_0000285 expression was more than three times higher in radiotherapy-resistant patients. Similarly, the saliva of OSCC patients showed increased levels of circ_0001874 and circ_0001971 [82].

Although RNA-based liquid biomarkers have received attention for recurrence/therapy response monitoring in several cancer types [83,84,85], their use in HNSCC recurrence monitoring has so far not been explored.

## 4. Discussion

With high rates of recurrent disease, the five-year relative survival of HNSCC has reached 67% [8]. Treatment of HNSCC, particularly in the relapsed setting, remains challenging [86]. Both existing and novel treatment modalities will be improved by the ability to make biomarker-led treatment decisions. Early detection of recurrent disease, even before any clinical or imaging-based signs, would allow treatment to begin before any frank relapse, and prepare for second (or higher)-line treatment decisions.

Since the most predominant oncogenic virus in head and neck cancer is HPV, several proofs-of-concept have been published on longitudinal measurements of HPV ctDNA in the saliva and/or plasma of HNSCC patients, demonstrating success in identifying tumor recurrence months earlier than other surveillance techniques [39,41,43,44,46]. EBV ctDNA is another promising biomarker with low cost and high sensitivity for predicting disease recurrence [46]. For non-viral-driven subtypes of HNSCC, specific mutations, methylation markers, or RNA expression have been explored as biomarkers [24,26,50,51,52,53,56,57,64,67,68,69,79,80,81,82].

Recurrence detection by means of liquid biopsy definitely has several advantages, including the possibility of rapid initiation of second-line treatments, which is potentially associated with improved outcomes and increased survival chances. In addition, rapid recurrence detection may further aid in the usage of personalized medicine approaches, which are quickly gaining interest in the clinics [87]. Nevertheless, the disadvantages of recurrence testing using liquid biopsies should be noted. Indeed, early detection options pose a challenge to doctors, as there is no targeted therapy suitable or available for each patient. Certainly, further research is needed to identify additional drugs ensuring advances in personalized medicine for HNSCC patients. Besides, as no extensive studies have been yet performed, clear guidance on the risk (progression and late-effects)—benefit ratio for liquid biopsy treatment-informed decisions is currently lacking. An added important consideration resulting from liquid biopsy-based monitoring is the psychological burden added to the patient, as intense monitoring may be a factor contributing to anxiety [88].

Despite these developments in liquid biopsy analysis, there is still a lack of implementation in clinical practice. This is in part due to the wide variability in methods used and often-small sample sizes, or a focus on a very specific subset of patients. Although there is a validated HPV ctDNA blood test available for the detection of HPV-driven cancer, it is currently available only in the United States (https://naveris.com/what-is-navdx/ accessed on 23 December 2021). Furthermore, the few treatment options available for when the tumor is not yet detectable by imaging techniques also limit the clinical implementation of these biomarkers. An integrated approach, using a panel of markers in both blood and saliva, might be the optimal way to predict recurrence with the highest sensitivity and specificity. Finally, very few authors have reported on the cost-effectiveness of liquid biopsies in clinical practice [89]. All of these factors will need careful consideration in future trial designs seeking to evaluate the use of liquid biopsies in HNSCC surveillance.

In conclusion, the studies highlighted here clearly indicate that liquid biopsy-based, non-invasive biomarkers can play an essential role in the detection and management of early recurrence of HNSCC. Further standardization and exploration of novel treatment options will be essential to unlocking the full potential of these fluid-based biomarkers.

## Data Availability

Not applicable.
